# Gratitude and academic engagement: exploring the mediating effects of internal locus of control and subjective well-being

**DOI:** 10.3389/fpsyg.2023.1287702

**Published:** 2023-12-20

**Authors:** Hongbo Cui, Xiaoyan Bi, Weiyu Chen, Tao Gao, Zaihua Qing, Keke Shi, Yankun Ma

**Affiliations:** ^1^School of Education, Guangzhou University, Guangzhou, China; ^2^Mental Health Education and Counseling Center, Guangzhou University, Guangzhou, China

**Keywords:** gratitude, academic engagement, internal locus of control, subjective well-being, mediating effects

## Abstract

This study aimed to explore the relationship between gratitude and academic engagement in Chinese students. The students of some junior high schools in Guangzhou were surveyed using the Gratitude Questionnaire-6, the School Engagement Questionnaire, the Levenson’s IPC Scale, and the General Well-being Schedule. A total of 708 valid responses were collected. The results indicate a significant positive relationship between gratitude and academic engagement. Subjective well-being plays a mediating role between gratitude and academic engagement. Locus of control and subjective well-being serve as serial mediators between gratitude and academic engagement. These findings suggest that promoting students’ academic engagement can be achieved by fostering gratitude and improving their internal locus of control and subjective well-being. By cultivating gratitude and enhancing these factors, educators and policymakers can create a more engaging and supportive learning environment for students.

## Introduction

Learning psychological problems such as learning anxiety and academic burnout often occur (Diestel and Schmidt, 2010; Fernández-Castillo, 2021), and improving the enthusiasm of adolescents to learn has always been a concern for educators. With the development of the positive psychology research paradigm, some researchers have shifted their attention from learning burnout to academic engagement in order to positively intervene in the learning behavior of adolescents. Academic engagement refers to an individual’s sustained cognitive, behavioral, and positive emotional state during the learning process (Skinner and Belmont, 1993), which reflects a high level of energy and a strong sense of identity in learning, and energy concentration without slack (Schaufeli et al., 2002). It can be achieved through positive emotions, a tendency to strive for a goal, and energetic behavior. It is valuable and urgent to study academic engagement as a multifaceted structure and to integrate emotional, cognitive, and behavioral inputs, as this depicts a richer picture of children than can be portrayed by a single component (Fredricks et al., 2004; Appleton et al., 2008). Academic engagement is an important indicator of education quality and student development. For example, engaged students are more likely to be motivated, persistent, and achieve higher levels of academic success than their less engaged peers (Fredricks et al., 2004). However, research has shown that many students struggle with academic engagement, which can lead to academic disengagement, lower academic performance, and even dropping out of school (Kiuru et al., 2014). Given the importance of academic engagement, it is essential to understand the factors that contribute to it.

Additionally, the high school stage is a critical period for the physical, mental, and intellectual development of individuals. Adolescents in this stage are experiencing rapid self-awareness growth, characterized by a combination of immaturity and maturity, which makes them more susceptible to environmental influences that can impact their academic engagement (Kiuru et al., 2014; Chen et al., 2015). Moreover, in China, junior high school students face significant pressure from the College Entrance Examination, leading to a heavy academic workload and challenging content. It becomes even more crucial for high school students to maintain a state of high academic engagement (Chen et al., 2015). Therefore, it is of great practical significance to study the influence mechanism of Chinese high school students’ academic engagement.

## Gratitude and academic engagement

The factor that has received increasing attention in recent years that affects academic engagement is gratitude. Gratitude is the positive emotions induced by positive subjective experiences and the positive cognition and personality acquired by others (Emmons and Shelton, 2005; Froh et al., 2010). Gratitude includes two categories: trait gratitude and state gratitude (Emmons and McCullough, 2003). We study the trait gratitude in this paper. Trait gratitude refers to the positive personality traits in which individuals experience gratitude in return for helping others, and from this deriving a positive personality trait of gratitude for daily life (Wood et al., 2008), which are important resilience factors for individual growth. Most scholars view gratitude as a positive personality trait with stability and consistency across time and context (Wood et al., 2008; He et al., 2013). Many psychologists have put forward theories to illustrate the important value of gratitude in human development, and empirical studies have shown that gratitude can promote the development of physical health, quality of life, mental resilience, and prosocial behavior in individuals (Watkins et al., 2003; Emmons and Shelton, 2005). Research has also found that gratitude reduces learning burnout in adolescents (Emmons and Shelton, 2005). Academic engagement, as the antithesis of burnout, may also be affected by gratitude. Recent studies have also suggested that gratitude may be associated with academic engagement. For example, a study by Froh et al. (2011) found that higher levels of gratitude were associated with higher levels of academic motivation and engagement among high school students. Similarly, Wen et al. (2010) found that gratitude can positively predict the academic engagement of Chinese high school students. In addition, the theory of psychological capital emphasizes the importance of individuals’ positive psychological traits for learning and work performance (Luthans et al., 2006; Luthans and Youssef-Morgan, 2017). Gratitude, as a positive emotion and attitude, can be considered as part of psychological capital and has a positive impact on academic engagement (Froh et al., 2010). When individuals have higher levels of psychological capital, including a sense of gratitude, they are more likely to actively engage in and participate in academic tasks. Therefore, gratitude, as part of psychological capital, can promote individuals’ academic engagement and improve academic outcomes.

However, there are some limitations in the above studies. First of all, most of the studies are carried out in the context of Western culture, and gratitude under Chinese Confucian culture and gratitude under Western Christian culture are quite different, so it is necessary to test the cultural similarity and specificity of gratitude. Secondly, gratitude is a traditional virtue of the Chinese nation, and ancient teachings such as “The gratitude of dropping water should be reciprocated by springs” still deeply influence Chinese today (Ma et al., 2022), but the study of gratitude in Chinese adolescents’ learning behavior is insufficient. In the context of collectivist cultures, gratitude is strongly associated with a sense of indebtedness that motivates the recipient to have an obligation to give back to the donor or others (Naito et al., 2005). In theory, students who feel more favored by others and show more gratitude will be more willing to stay in school, more receptive to their teachers’ views, and study harder. In fact, Wen et al. (2010) also verified this hypothesis: gratitude in high school students can positively predict their academic engagement. Despite these findings, the mechanisms underlying the relationship between gratitude and academic engagement among Chinese high school students are not well understood. In particular, it is unclear whether gratitude operates through specific psychological processes that mediate the relationship between gratitude and academic engagement.

## Internal locus of control as a mediator

One such psychological process that mediate the relationship between gratitude and academic engagement may be internal locus of control. The concept of Locus of Control (LOC) has been written about extensively since the term was first introduced by Rotter. LOC refers mainly to people’s general perception of the outcome of a behavior or event, which is determined by expectations and reinforcement values in a particular context (Rotter, 1966). There are differences between internal control and external control in the source of individual psychological control, and internally controlled individuals usually believe that the development of things is determined by their own behavior, personality and ability, while external controllers believe that the development of things is mainly determined by external forces and is less affected by individual behavior (Guo, 2000). Locus of control and a person’s position along a bipolar scale of internalism to externalism has been shown to have effect on many aspects of one’s life (Thomas, 2013). Previous research has demonstrated that individuals with strong internal locus of control are more likely to engage in academic pursuits and achieve academic success (Grillo, 2015; Dong et al., 2017). Specifically, students with high internal locus of control tend to attribute academic performance to their own controllable factors, so they are more likely to actively participate in learning through subjective initiative to change the unfavorable situation and strive for better results. According to the self-determination theory (SDT) (Ryan and Deci, 2000), individuals’ motivation for their behavior stems from intrinsic autonomy and perceived (internal locus of control) importance. A sense of gratitude can enhance the intrinsic motivation of junior high school students toward learning, making them more willing to invest time and energy in completing academic tasks, thereby demonstrating higher academic engagement (Singh et al., 2021).

On the other hand, several studies have explored the relationship between gratitude and internal locus of control. In a study by Emmons and McCullough (2003) participants who were asked to write down things they were grateful for over a period of 10 weeks reported an increase in their internal locus of control beliefs, indicating that gratitude may have a positive impact on an individual’s belief in their ability to control events in their lives. Another study by Ping and Peng (2018) found that people with gratitude traits have a higher level of inner control and a sense of control and autonomy over their lives. Similarly, Duprey et al. (2018) found that gratitude interventions increased perceptions of control in both daily life and stressful situations, suggesting that gratitude can enhance an individual’s belief in their ability to cope with challenging situations. Therefore, we hypothesized that students with high levels of gratitude may have high internal locus of control and, in turn, may show more academic engagement.

## Subjective well-being as a mediator

Subjective Well-Being (SWB) mainly refers to people’s overall evaluation of their quality of life, including three dimensions: life satisfaction, positive emotions and negative emotions (Diener et al., 2015). At the individual level, subjective well-being measures an important aspect of an individual’s mental health (Yao et al., 2018) and is also an important indicator of the positive degree of an individual’s psychological development (Donaldson et al., 2015). Subjective well-being may also have a certain impact on academic engagement. For example, Diestel and Schmidt (2010) suggested that individuals with higher levels of subjective well-being tend to be more resilient in the face of challenges, which may translate to greater academic engagement. Similarly, research has shown that students with higher levels of subjective well-being tend to have better academic outcomes, such as higher grades and greater persistence in the face of academic challenges (Suido et al., 2008).

Besides, according to the Broaden-and-Build theory of Positive Emotions (Isgett and Fredrickson, 2004), gratitude can broaden people’s thinking patterns and construct positive social and psychological resources, thereby enhancing individual subjective well-being. Empirical researches also explored the important role of gratitude in subjective well-being. Previous studies have shown a positive correlation between gratitude and subjective well-being (Yoshimura and Berzins, 2017). Longitudinal studies have also found that gratitude is a predictor of subjective well-being (Luo and Zhou, 2015).

Moreover, Severino et al. (2011) suggested that individuals with a stronger internal locus of control may experience more positive emotions, such as pride and satisfaction, as a result of their sense of control over their own outcomes. These positive emotions, in turn, contribute to greater subjective well-being overall. A recent study also found similar results that internally controlled individuals are more likely to attribute success to their own efforts and abilities, which can lead to subjective well-being (Smallwood et al., 2023).

Given these findings, it is possible that both internal locus of control and subjective well-being may mediate the relationship between gratitude and academic engagement. In addition, China is a collectivistic culture that places a strong emphasis on filial piety, respect for authority, and group harmony (Hofstede, 2001). These cultural values may influence the way in which gratitude is experienced and expressed, as well as the extent to which gratitude is associated with academic engagement. Therefore, this study aims to investigate the relationship between gratitude and academic engagement in a Chinese context, and to examine the mediating roles of internal locus of control and subjective well-being in this relationship. In the present study, we hypothesize that internal locus of control and subjective well-being may act as parallel mediators, with each pathway independently mediating the relationship between gratitude and academic engagement. Additionally, internal locus of control and subjective well-being may act as a chain mediator, with gratitude predicting internal locus of control, which in turn predicts subjective well-being, and ultimately predicts academic engagement. This means that gratitude may predict internal locus of control, which in turn predicts subjective well-being, and ultimately leads to academic engagement. By conducting this study within a Chinese cultural context, we can gain insights into how cultural values and beliefs impact the relationship between gratitude and academic engagement. This research has the potential to contribute to both the existing literature on gratitude and academic engagement, as well as provide a deeper understanding of the cultural factors that shape these associations in a collectivistic society like China.

**Figure 1:**
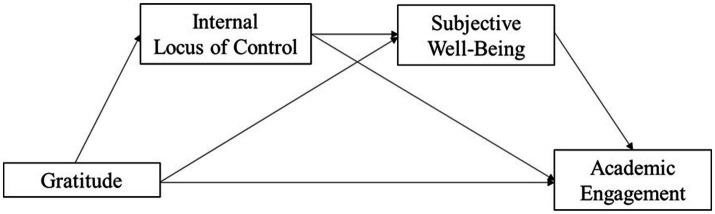
The hypothesized model.

Based on the above, this study proposes the following three hypotheses: (1) Gratitude has a positive predictive effect on academic engagement; (2) internal locus of control and subjective well-being serve as parallel mediators between gratitude and academic engagement; (3) internal locus of control and subjective well-being also serve as a chain mediating between gratitude and academic engagement. The model is illustrated in Figure 1.

## Materials and methods

### Participants

This study used convenience sampling and questionnaires of the study were gathered in March 2022 in certain normal high schools in Guangdong Province in China. After obtaining the informed consent of the school leaders and students, the researchers first explained the rules for filling in the questionnaire to the students, and then the students completed the questionnaire through mobile phones independently. A total of 724 questionnaires were distributed. After eliminating incompleteness questionnaires, 708 valid questionnaires were obtained, with an effective rate of 97.79%. The average age of the subjects is 16.47 ± 1.01 years. Among them, there are 318 (44.92%) males, 372 (55.08%) females; 228 (32.20%) only children, and 462 (65.25%) non only children. The number and percentage of students in junior high school from first to third year are 106 (14.97%), 520 (73.45%) and 82 (11.58%) respectively.

## Measures

### Gratitude Questionnaire-6

Gratitude was measured using the Gratitude Questionnaire-6 (GQ-6). The GQ-6 consists of six items that assess an individual’s dispositional gratitude. Responses are rated on a 7-point Likert scale ranging from 1 (strongly disagree) to 7 (strongly agree). Example items include “I have so much in life to be thankful for” and “If I had to list everything that I felt grateful for, it would be a very long list.” Items 3 and 6 are reverse scoring questions. Higher scores indicate a higher level of gratitude. The questionnaire was modified by Wei et al. (2011) for localization to be suitable for Chinese. The Chinese version has been reported as a reliable tool. The GQ-6 Cronbach’s α coefficient is 0.81 in the present study.

### Levenson’s IPC scale

Levenson’s IPC scale is a commonly used tool for measuring locus of control, including three dimensions: internal, powerful others and chances (Levenson, 1973). Internal locus of control was assessed by the Internality subscale from the Levenson’s IPC Scale. The examples of items are: “When I make plans, I am almost certain to make them work”; “When I get what I want, it is usually because I worked hard for it.” The Levenson’s subscale consists of eight items that are rated on a 6-point Likert scale from −3, indicating complete disagreement, to 3, indicating complete agreement. When calculating the score, 24 is added to offset the negative score, so each subscale score is 0–48. The Levenson’s subscale Cronbach’s α coefficient was 0.75 in the present study.

### General well-being schedule

The General Well-being Schedule (GWB) was used to measure subjective well-being. The GWB was developed by the National Center for Health Statistics in the United States as a prescriptive testing tool to evaluate subjective feelings of happiness among subjects. There are 33 items in the scale, and the higher the score, the higher the level of subjective well-being. This Chinese version revised by Duan (1996) consists of 18 items for evaluation. Responses are rated on a 7-point Likert scale ranging from 1 (strongly disagree) to 7 (strongly agree). The GWB Cronbach’s α coefficient was 0.77 in the present study.

### School engagement questionnaire

The School Engagement Questionnaire (SEQ) developed by Wang et al. (2011) was used to measure academic engagement. The SEQ consists of 23 items that assess an individual’s cognitive, behavioral, and emotional engagement in learning activities. Responses are rated on a 5-point Likert scale ranging from 1 (strongly disagree) to 5 (strongly agree). The SEQ includes three dimensions of behavioral engagement, emotional engagement, and cognitive engagement, which can better reflect various aspects of academic engagement. The higher the score, the higher the level of academic engagement. In this study, the Chinese version of the SEQ Cronbach coefficient was 0.83.

### Data analysis and common method bias test

This study used SPSS 26.0 to perform descriptive statistics, *t*-test, and correlation analysis on the collected data, and used the PROCESS V4.0 macro program of Hayes to test and analyze mediating effects. The data collected in this study is self-reported, so the common method deviation test is required. In this study, Harman single factor test method was used for exploratory factor analysis. The results showed that 11 common factors with characteristic value greater than 1 were obtained from the factor analysis without rotation, and a total of 11 factors were greater than 1. The first factor explained 17.03% variance and less than 40% marginal value, indicating that there was no serious common method deviation in this study (Zhou and Long, 2004).

## Results

### Descriptive statistics and correlation analysis

Descriptive statistics and Pearson’s correlations for the main variables are presented in Table 1. The results show gratitude is positively correlated with internal locus of control, subjective well-being, and academic engagement, respectively (*p* < 0.001). Internal locus of control is positively correlated with subjective well-being and academic engagement, respectively (*p* < 0.001). Subjective well-being is significantly positively correlated with academic engagement (*p* < 0.001). In addition, *t*-tests were conducted to examine the differences between variables. The results revealed that males had significantly higher levels of internal locus of control compared to females (*t* = 4.96***). However, no significant differences were found for the other variables.

**Table 1:** Descriptive statistics and correlation coefficient matrix (*N* = 708).

	Male	Female	1	2	3	4
1 Gratitude	5.07 ± 1.12	5.04 ± 1.09	1			
2 Internal locus of control	30.57 ± 8.46	27.17 ± 9.11	0.39^***^	1		
3 Subjective well-being	79.32 ± 11.67	77.72 ± 11.55	0.44^***^	0.44^***^	1	
4 Academic engagement	79.99 ± 13.37	81.06 ± 12.68	0.41^***^	0.18^***^	0.42^***^	1

^**^p < 0.01, ^***^p < 0.001.

### Regression and mediation effect analysis

According to the results of the correlation analysis in this study and the statistical preconditions of the mediation effect, further mediation effect analysis of internal locus of control and subjective well-being can be carried out (Wen and Ye, 2014). With gratitude as the independent variable, academic engagement as the dependent variable, internal locus of control and subjective well-being as the intermediary variables, the study used the bias-corrected percentile Bootstrap method in the SPSS macro program Process compiled by Hayes to analyze the mediating effect, and Model 6, which specialized in analyzing chain mediation effects, was used for testing. The Bootstrap sampling number is 5000, the confidence interval is set to 95%.

The results showed that gratitude has a positive predictive effect on academic engagement (*β* = 0.30, *p* < 0.001); gratitude has a positive predictive effect on internal locus of control (*β* = 0.39, *p* < 0.001); gratitude has a positive predictive effect on subjective well-being (*β* = 0.31, *p* < 0.001); subjective well-being has a positive predictive effect on academic engagement (*β* = 0.32, *p* < 0.001); internal locus of control has a positive predictive effect on subjective well-being (*β* = 0.32, *p* < 0.001). Internal locus of control had no positive predictive effect on academic engagement (*β* = −0.08, *p* > 0.05) (see Table 2).

**Table 2:** Model for regression analysis between variables (*N* = 708).

Predictor variable	Outcome variable: ILC		Outcome variable: SWB		Outcome variable: AE	
	*β* (Boot SE)	95% Boot CI	*β* (Boot SE)	95% Boot CI	*β* (Boot SE)	95% Boot CI
Gr	0.39 (0.05)	[0.43, 0.61]	0.31 (0.06)	[0.44, 0.68]	0.30 (0.08)	[0.44, 0.75]
ILC			0.32 (0.04)	[0.33, 0.51]	−0.08 (0.06)	[−0.23, 0]
SWB					0.32 (0.04)	[0.28, 0.46]
	*R*^2^ = 0.15		*R*^2^ = 0.28		*R*^2^ = 0.24	
	*F* = 121.08***		*F* = 133.62***		*F* = 71.80***	

**p < 0.01, ***p < 0.001. Gr, Gratitude; ILC, Internal Locus of Control; SWB, Subjective Well-Being; AE, Academic Engagement.

**Figure 2:**
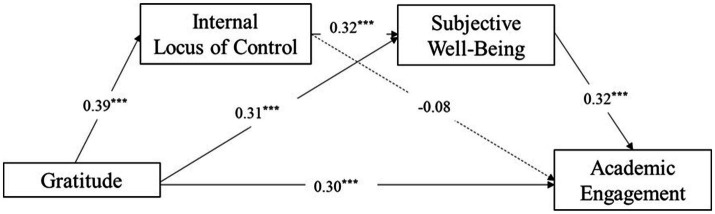
Mediating effects of internal locus of control and subjective well-being. **p < 0.01, ***p < 0.001.

The intermediary effect test showed that the direct effect of gratitude on academic engagement was 0.41, accounting for 74.54% of the total effect; gratitude affected academic engagement through subjective well-being, and the intermediary effect was 0.10, accounting for 18.18% of the total effect of family function on altruistic behavior; gratitude had an impact on academic engagement through the chain mediating roles of internal locus of control and subjective well-being, with an effect of 0.04, accounting for 7.27% of the total effect. The mediating effect of internal locus of control between gratitude and academic engagement was not significant (The 95% Boot CI is [−0.07, 0.01], including 0) (see Table 3 and Figure 2).

**Table 3:** Mediation effect analysis (*N* = 708).

Model pathways	Effect size	Boot SE	Boot LLCI	Boot ULCI
Gr → AE	0.41	0.07	0.68	0.95
Gr → ILC → AE	−0.03	0.02	−0.07	0.01
Gr → SWB → AE	0.10	0.02	0.07	0.14
Gr → ILC → SWB → AE	0.04	0.01	0.02	0.06

Gr, Gratitude; ILC, Internal Locus of Control; SWB, Subjective Well-Being; AE, Academic Engagement.

## Discussion

To summarize the findings, the results suggested that gratitude has significant positive predictive effect on academic engagement, which is consistent with previous research (Wen et al., 2010; Froh et al., 2011). Basic self-determination theory, states that everyone has three innate psychological needs: autonomy, competence, and relatedness. A sense of gratitude can enhance the intrinsic motivation of junior high school students toward learning, making them more willing to invest time and energy in completing academic tasks (Ryan and Deci, 2000). In empirical research, Kashdan et al. (2010) found that the key factor behind gratitude is the awareness of various good deeds, which can raise people’s self-confidence and sense of autonomy. When these three basic psychological needs are satisfied, the internal motivation of individuals is stimulated and they show stronger initiative, enthusiasm, and persistence in their study (Neufeld et al., 2020). Therefore, students with a high level of gratitude are more likely to be satisfied with their basic psychological needs, thereby demonstrating more academic engagement.

This study further found that gratitude plays a role in academic engagement through various paths. The gratitude affected academic engagement behavior through subjective well-being. The broaden-and-build theory of positive emotions (Isgett and Fredrickson, 2004; Fredrickson, 2013) believes that positive emotions can expand the attention and cognitive range of individuals, stimulate the flexibility and creativity of thinking, promote individuals to more effectively acquire and analyze information, continuously acquire knowledge and experience conducive to achieving goals, stimulate new problem-solving strategies, and construct persistent resources (intellectual and psychological resources). It is hypothesized that students with high subjective well-being have a greater attention span and cognitive range during the learning process, and their thinking is more flexible and creative. They can effectively master learning methods, accumulate learning experience, provide favorable conditions for learning, and maintain a good learning state. At the same time, positive emotions can enable students to experience a better sense of control, making them more willing to actively construct knowledge, and more engaged in learning (D'Errico et al., 2016). On the other hand, gratitude is closely related to positive appraisals of self and life, and can lessen negative emotions (Magno and Orillosa, 2012). Positive life evaluation, more positive emotions, and less negative emotions are important dimensions of subjective well-being (Ping and Peng, 2018). Therefore, gratitude can enhance individual subjective well-being, which can widen and strengthen social, intellectual and psychological resources, which help individuals demonstrate more academic engagement. While trait gratitude is generally associated with well-being and positive relationships, it is important to note that expressing gratitude inappropriately or excessively can sometimes lead to feelings of indebtedness or dependency. Expressing gratitude inappropriately or excessively may lead to feelings of indebtedness or dependency in the recipient. If gratitude is expressed in a way that creates an imbalanced power dynamic or places an excessive burden on the recipient, it can potentially lead to feelings of obligation or pressure. This can hinder the authenticity and genuine nature of the gratitude exchange and may even strain relationships. Additionally, if gratitude is expressed excessively, it may lose its impact and become less meaningful over time. This can result in a diminished sense of appreciation and potentially undermine the positive effects associated with trait gratitude. Therefore, it is essential for individuals to be mindful of how they express gratitude and consider the appropriateness and balance in their interactions. By being sensitive to the dynamics of gratitude expression, individuals can ensure that it remains a genuine and positive experience for both the giver and the recipient, fostering well-being and strengthening relationships.

Besides, internal locus of control between gratitude and academic engagement was not significant. There are several potential reasons for this non-significant finding. It is possible that other factors not considered in our study may have influenced the relationship between gratitude and academic engagement. Additionally, the sample size and specific characteristics of the participants in our study may have impacted the results. Further research is necessary to explore these factors and better understand the underlying mechanisms between gratitude, internal locus of control, and academic engagement.

This study also found that gratitude could influence academic engagement through a chain-mediated effect of internal locus of control and subjective well-being. This suggested that gratitude may be associated with greater academic engagement in part because it fosters a sense of personal control and a positive outlook on life. Prior studies have mostly analyzed the relationship between gratitude, internal locus of control, subjective well-being, and academic engagement. For example, Grillo (2015) showed that individuals who believe they have greater control over their lives are more likely to engage in behaviors; Suido et al. (2008) found that subjective well-being can significantly predict academic engagement. This study is the first systematic analysis of the chain mediating effects of internal locus of control and subjective well-being on the relationship between gratitude and academic engagement. According to the basic psychological needs, gratitude helps people make choices that meet their own basic psychological needs and makes them more confident in their own abilities, thereby causing them to experience a greater sense of internal locus of control (Hamarta et al., 2013; Cazan and Dumitrescu, 2016). Furthermore, according to the locus of control (Rotter, 1966), internal locus of control is considered as promoting factors for individual mental health and development. Individuals with high internal locus of control believe that they can control their own lives and that their actions can change their current situation. On the one hand, their sense of control over events makes it easier for them to feel their own strength and value, and thus have a higher sense of life significance (Ye and Lin, 2015). On the other hand, in the face of stressful life events, individuals with internal locus of control are more likely to have stable emotions and a higher sense of self-efficacy, and exhibit stronger psychological resilience (Buddelmeyer and Powdthavee, 2016). According to the theory of psychological capital, individuals with high psychological capital, tend to have more flexible cognition and richer coping strategies (Tugade et al., 2005), which can help reduce negative emotional distress and increase positive emotions, thereby positively predicting individual subjective well-being. According to the broaden-and-build theory of positive emotions indicates (Fredrickson, 2013), students with high subjective well-being are often able to actively adjust learning strategies, devote more psychological resources to learning, effectively monitor their learning behavior, and maintain a high level of academic engagement (Yao et al., 2018). Thus, this finding indicated that individuals with high levels of gratitude tend to have a greater sense of control over their own lives; individuals who have a sense of control over their own lives are further more likely to experience higher subjective well-being, which can translate into greater academic engagement.

However, this study has several limitations. Firstly, the use of a cross-sectional design restricts our ability to establish causal relationships between gratitude, academic engagement, internal locus of control, and subjective well-being. This design captures data at a single time point, making it difficult to determine the directionality of the observed relationships. It is possible that these relationships are bidirectional or influenced by unmeasured confounding variables. Secondly, the use of convenience sampling in this study may introduce limitations and affect the generalizability of the findings. By selecting participants based on their easy availability in certain normal high schools in Guangdong Province in China, the sample may not accurately represent the entire population of high school students in the region or the broader population. Therefore, caution should be exercised when generalizing the study’s findings beyond the specific sample and context. Third, reliance on self-report measures to assess variables such as gratitude, academic engagement, internal locus of control, and subjective well-being introduces the potential for biases. Self-report measures are susceptible to social desirability bias, where participants may provide responses that they perceive as socially desirable, rather than reflecting their true experiences or thoughts. Additionally, response bias may occur, leading to inaccuracies in the collected data. It is important to consider these limitations when interpreting the findings of the study. Future research should employ longitudinal designs and incorporate objective measures to establish causal relationships and reduce biases associated with self-report measures. Additionally, in order to address the limitations of convenience sampling in this study, future prospects include the use of more rigorous sampling techniques. Researchers can consider implementing probability sampling methods like stratified random sampling or cluster sampling. These methods offer greater control over sample characteristics and increase the likelihood of obtaining a representative sample. By adopting these techniques, researchers can enhance the validity and generalizability of their findings. Finally, utilizing diverse samples and controlling for potential confounding variables can enhance the generalizability and validity of the results.

## Conclusion

The present study provides evidence for the positive relationship between gratitude and academic engagement, and suggests that this relationship is partially explained by internal locus of control and subjective well-being. These findings have important implications for educational practice, suggesting that gratitude interventions may be a promising approach to promoting academic engagement and success. Future research should aim to replicate and extend these findings, and to explore potential moderators of the observed relationships. By gaining a deeper understanding of the factors that promote academic engagement, educators and policymakers can better support student success and well-being.

## Data availability statement

The raw data supporting the conclusions of this article will be made available by the authors, without undue reservation.

## Ethics statement

The studies involving humans were approved by Research Ethics Committee of Guangzhou University. The studies were conducted in accordance with the local legislation and institutional requirements. Written informed consent for participation in this study was provided by the participants’ legal guardians/next of kin.

## Author contributions

HC: Conceptualization, Data curation, Formal analysis, Investigation, Writing – original draft, Methodology, Validation, Visualization. XB: Conceptualization, Data curation, Formal analysis, Investigation, Methodology, Software, Writing – original draft. WC: Data curation, Investigation, Methodology, Software, Writing – review & editing. TG: Data curation, Investigation, Methodology, Supervision, Writing – review & editing. ZQ: Data curation, Investigation, Methodology, Validation, Writing – review & editing. KS: Data curation, Investigation, Validation, Visualization, Writing – review & editing. YM: Conceptualization, Funding acquisition, Resources, Supervision, Writing – review & editing.
